# Application of Targeted and Suspect Screening Workflows for Cyclic Peptide Cyanotoxin Profiling in Spirulina- and Klamath-Based Food Supplements

**DOI:** 10.3390/foods14172969

**Published:** 2025-08-26

**Authors:** Laura Carbonell-Rozas, M. Mar Aparicio-Muriana, Roberto Romero-González, Antonia Garrido Frenich, Ana M. García-Campaña, Monsalud del Olmo-Iruela

**Affiliations:** 1 Department of Analytical Chemistry, University of Granada, Av. Fuente Nueva s/n, 18071 Granada, Spain; mmaraparicio@ugr.es (M.M.A.-M.); amgarcia@ugr.es (A.M.G.-C.); mdolmo@ugr.es (M.d.O.-I.); 2 Department of Chemistry and Physics, Research Centre for Mediterranean Intensive Agrosystems and Agrifood Biotechnology (CIAIMBITAL), Agrifood Campus of International Excellence (ceiA3), University of Almeria, 04120 Almeria, Spain; rromero@ual.es (R.R.-G.); agarrido@ual.es (A.G.F.)

**Keywords:** cyanotoxins, food supplements, blue-green algae, food safety, UHPLC-MS/MS, suspect screening

## Abstract

Spirulina (*Arthrospira* spp.) and klamath (*Aphanizomenon flos-aquae*) are widely consumed cyanobacteria-based food supplements valued for their nutritional and health-promoting properties. However, these products are susceptible to contamination by cyanotoxins, which are potent toxins produced by co-occurring cyanobacteria that may pose health risks to consumers. In this study, we applied an integrated targeted and suspect screening approach to comprehensively assess the presence of cyanotoxins in commercial spirulina- and klamath-based food supplements. Targeted analysis was performed using UHPLC-QqQ under dynamic multiple reaction-monitoring conditions optimized for the determination of twelve cyclic peptide cyanotoxins. Suspect screening was conducted using high-resolution mass spectrometry (HRMS) with a Q-Orbitrap analyser, applying a specific workflow to detect additional related compounds lacking analytical standards. The method enabled the detection and identification of multiple cyanotoxins, including microcystins, nodularin, and anabaenopeptins. The combination of targeted and suspect workflows allowed for a broader coverage of potential related cyanotoxins. Several cyanotoxins were detected in a klamath-based supplement, with high concentrations of microcystin-RR, while additional variants were identified through suspect screening. These findings highlight the need for routine monitoring and stricter regulatory oversight of cyanobacteria-based supplements to ensure consumer safety.

## 1. Introduction

In recent years, spirulina (*Arthrospira* spp.) and klamath (*Aphanizomenon flos-aquae*, or AFA) have gained significant popularity worldwide as food supplements, promoted for their high nutritional value and purported health benefits [1]. These cyanobacteria are rich in proteins, essential amino acids, vitamins, minerals, and bioactive compounds. They are widely consumed by health-conscious individuals and are often marketed as “superfoods” [2]. Spirulina is particularly noted for its high concentration of phenols, phycocyanin pigment, and polysaccharides, which all take part in a number of biological activities, showing antioxidant and anti-inflammatory properties [3]. On the other hand, klamath is known for containing phenylethylamine (PEA), a naturally occurring trace amine that acts as a neuromodulator in the central nervous system. PEA has been associated with mood regulation and cognitive enhancement, though its stability and bioavailability in supplement form are still under investigation [4]. Both are filamentous cyanobacteria, commonly referred to as blue-green algae (BGA), which grow in freshwater environments. Spirulina is typically cultivated in controlled aquaculture systems, while klamath is harvested from natural sources, particularly the Upper Klamath Lake in Oregon (USA), where it grows seasonally in large blooms.

The increasing demand for nutrient-rich functional food products such as spirulina and klamath is reflected in current market trends. According to a report by Credence Research, the global algae products market is projected to grow from USD 5291.84 million in 2023 to approximately USD 9728.83 million by 2032, representing a compound annual growth rate (CAGR) of 7.00% between 2024 and 2032 [5]. Among the cyanobacterial species used in commercial products, *Arthrospira platensis* and *Arthrospira maxima* (commonly marketed as spirulina) are the most important in terms of commercial production and sales [6].

However, despite their natural origin and nutrient profile, both spirulina- and klamath-based products are susceptible to be contaminated by cyanotoxins, which are toxic secondary metabolites produced by various cyanobacterial species that may coexist in the same aquatic environments or emerge during bloom events. Cyanotoxins such as microcystins (MCs), cylindrospermopsin (CYN), anatoxin-a (ANA), and saxitoxins (SXTs) have been associated with serious health effects in humans, including hepatotoxicity, endocrine disruption effects, neurotoxicity, and potential carcinogenicity [7,8,9]. While Arthrospira species are not toxic, as no direct production of toxins has been reported, klamath is known to be capable of producing toxins such as ANA, SXTs, CYN, and β-methylamino-L-alanine (BMAA) [10].

Cyclic peptides are a major class of cyanotoxins typically consisting of heptapeptides or pentapeptides and are among the most toxic and commonly detected. They include MCs, nodularins (NODs), and anabaenopeptins (APs) [11]. The presence of these toxins in spirulina- and klamath-based food supplements poses a significant public health concern, particularly in the absence of harmonized regulatory controls and routine testing protocols. Some regulations for cyanotoxins, particularly microcystin-LR (MC-LR), in drinking water have been established by organizations such as the World Health Organization (WHO) [12] and Health Canada [13]. The WHO has set a tolerable daily intake (TDI) of 0.04 µg/kg body weight (bw) for chronic exposure to MC-LR and recommends a maximum concentration of 1 µg/L in drinking water. However, specific legislation regulating their occurrence in food and dietary supplements remains lacking or inconsistent across regions. To date, the Oregon Health Division and the Oregon Department of Agriculture have established a regulatory limit of 1 μg/g for MCs in BGA-containing products [14].

In this context, a growing number of studies have reported the presence of cyanotoxins in BGA-food supplements [7,15,16], underscoring the need for improved monitoring. Among them, MCs are the most studied and known in such products because of their prevalence and toxicity. Analytical methodologies for cyanotoxin detection in algal food products have evolved considerably over the past two decades. Conventional techniques such as enzyme-linked immunosorbent assays (ELISAs) and liquid chromatography with ultraviolet or fluorescence detection (LC-UV/FLD) offer practical tools for preliminary screening [17,18]. However, these methods often lack selectivity and may not differentiate between structurally similar analogues. In contrast, liquid chromatography–tandem mass spectrometry (LC-MS/MS) has become the gold standard for the quantitative determination of well-characterized cyanotoxins, providing high sensitivity and selectivity suitable for regulatory and surveillance purposes [19]. Most of the proposed analytical methods are focused on a single family of compounds. Although hydrophilic interaction liquid chromatography coupled with tandem mass spectrometry (HILIC-MS/MS) has been recently proposed for multiclass cyanotoxin determination [20], the inherent limitation of targeted LC-MS/MS is its reliance on the availability of analytical standards. To overcome this limitation and considering that MCs are a family of almost 250 structurally similar hepatotoxins, suspect and non-targeted screening approaches using high-resolution mass spectrometry (HRMS) are increasingly being explored [21,22], with the recent implementation of ion mobility spectrometry [23]. These approaches enable the detection of both known compounds lacking commercial standards (suspect screening) and previously unreported cyanotoxins (non-target screening), utilizing in silico databases and spectral libraries. Despite its advantages and the growing concern over emerging and structurally diverse cyanotoxins, HRMS remains underutilized in the quality assessment of food supplements and requires further development and validation to enable its broader application in this context.

In this study, a combined targeted and suspect screening workflow was applied using UHPLC-MS/MS and HRMS to analyze a selection of spirulina- and klamath-based food supplements commercially available on the Spanish market. Our primary objective was to comprehensively profile cyclic peptide cyanotoxins, both known and suspect, as well as identify potential interconnections by molecular networks. This work contributes to the growing body of evidence supporting the need for advanced analytical strategies in the safety assessment of cyanobacteria-derived dietary products and the importance of international legislation to ensure product safety and protect consumer health.

## 2. Materials and Methods

### 2.1. Reagents and Materials

All solvents were of LC-MS grade unless otherwise specified. Methanol (MeOH) and ultrapure water were purchased from Honeywell (Morristown, NJ, USA). Formic acid (FA) was acquired from Sigma-Aldrich (Darmstadt, Germany). Analytical standards for cyanotoxins were purchased individually from Enzo Life Sciences, Inc. (Lausen, Switzerland). Standard stock solutions of 50 or 25 μg/mL were prepared by adding 1 mL of the desired solvent directly into the toxin’s vial supplied by the manufacturer and gently swirling the vial to dissolve the toxin. Microcystin–leucine–arginine (MC-LR), microcystin–tyrosine–arginine (MC-YR), microcystin–tryptophan–arginine (MC-WR), microcystin–leucine–alanine (MC-LA), microcystin–leucine–tyrosine (MC-LY), microcystin–leucine–tryptophan (MC-LW), microcystin–leucine–phenilalanine (MC-LF), microcystin–homoisoleucine–arginine (MC-HliR), microcystin–homotyrosine–arginine (MC-HtyR), [DAsp3] microcystin–leucine–arginine ([DAsp3]-MC-LR), anabaenopeptin A (ApA), and anabaenopeptin B (ApB) were prepared in 100% MeOH; nodularin (NOD) was prepared in MeOH:H_2_O (1:1, *v*/*v*); and microcystin–arginine–arginine (MC-RR) was prepared in MeOH:H_2_O (80:20, *v*/*v*). These stock solutions were used to prepare working solutions containing a mixture of all compounds at specific concentrations in MeOH:H_2_O (1:1, *v*/*v*). These solutions were stored at 4 °C and allowed to reach room temperature before use. Detailed information of the structure of target cyanotoxins can be found in Table S1 in the Supplementary Materials section.

Strata-X cartridges (200 mg) and CLARYFY polytetrafluoroethylene (PTFE) hydrophilic syringe filters (0.2 μm × 13 mm) supplied by Phenomenex (Torrance, CA, USA) were used for the sample treatment procedure.

### 2.2. Sample Collection and Sample Treatment

Dietary supplements based on BGA, specifically spirulina and klamath, were sourced from both local retail stores in Granada (Spain) and online retailers. In some cases, these products also contained microalgae Chlorella and the brown algae Fucus in different ratios. They were sold as tablets, capsules and powder. Detailed information on all samples with their form, suppliers, daily dose, and content is listed in Table S2. All samples were analyzed before their expiration date.

BGA-based food supplements were individually ground to a fine powder, and 75 mg was weighed into a 15 mL centrifuge tube. Then, 4 mL of MeOH:H_2_O (80:20, *v*/*v*) was added to the sample, followed by agitation using an immersion disperser for 5 min. Afterwards, the sample was centrifuged at 9000 rpm and 4 °C for 10 min, and the supernatant was carefully transferred to a 50 mL centrifuge tube. To achieve a solution containing 20% MeOH, deionized water was added to the supernatant, increasing its volume to 16 mL. This step was necessary to ensure effective retention of the target compounds in the SPE cartridge. This solution was then subjected to purification using SPE.

The SPE was conducted in a Strata-X cartridge (200 mg, 6 mL), which was conditioned and activated with 3 mL of MeOH followed by 3 mL of deionized water. Subsequently, the extraction solution was loaded by gravity, and the cartridge was washed with 2 mL of MeOH:H_2_O (30:70, *v*/*v*) and dried under a vacuum for 1 min. The elution was performed by gravity using 5 mL of MeOH. The eluate was evaporated to dryness under a gentle stream of nitrogen, and the residue was re-dissolved with 250 μL of MeOH:H_2_O containing 0.1% FA (20:80, *v*/*v*). The final extract was filtered through a CLARIFY-PTFE hydrophilic syringe filter (0.2 μm × 13 mm) and transferred to a 0.3 mL glass insert for subsequent LC analysis.

### 2.3. Targeted Analysis by UHPLC-MS/MS

Chromatographic analysis was carried out employing a 1290 RRLC liquid chromatograph instrument (Agilent, Santa Clara, CA, USA) with a Jet Stream electrospray ionization (ESI) source (G1958–65,138) and interfaced to an Agilent triple quadrupole (QqQ) mass spectrometer (6460 A). Chromatographic separation of target compounds was performed in a UPLC BEH C18 column (2.1 mm × 100 mm, 1.7 μm particle size) using a mobile phase consisting of water as eluent A and acetonitrile as eluent B, both containing 0.1% FA at a flow rate of 0.4 mL/min. The gradient program was established as follows: 0 min, 0% of eluent B; 7 min, 80% of eluent B; 7.1 min, 100% of eluent B; 11 min, 100% of eluent B; 11.1 min, 0% of eluent B; and 14 min, 0% of eluent B. The column temperature was set at 45 °C, the autosampler temperature was set at 10 °C, and 5 μL was used as the injection volume.

An electrospray ionization tandem mass spectrometry (ESI-MS/MS) system operating in positive and negative mode in dynamic multiple reaction-monitoring (dMRM) conditions was employed to determine the cyclic peptide cyanotoxins. The precursor ion was selected based on the highest signal intensity, and in all cases, two transitions were monitored for quantification and identification purposes. The source parameters were set as follows: drying gas temperature, 300 °C; drying gas flow, 5 L/min; sheath gas temperature, 345 °C; sheath gas flow, 11 L/min; nebulizer, 45 psi; capillary, 3500 V; and nozzle voltage, 500 V. Optimization of the UHPLC–MS/MS parameters was performed by direct infusion of individual standard solutions, each at 1 mg/L in MeOH and at a flow rate of 0.2 mL/min. The main dMRM parameters and retention time are given in Table 1.

The Agilent MassHunter Workstation and Agilent Mass Hunter Quantitative Analysis for QqQ software were employed for data acquisition and processing, respectively.

### 2.4. Target Method Characterization

The proposed HPLC-MS/MS method was evaluated considering several parameters including linear range, sensitivity, limit of detection (LOD) and limit of quantification (LOQ), extraction efficiency, precision (i.e., repeatability and intermediate precision), and matrix effects (MEs). A spirulina sample that showed no detectable levels of the target compounds and no interference from endogenous substances with the analytes’ retention times was used as a representative blank matrix for method characterization.

Matrix-matched calibration curves were established in the representative spirulina samples, which were subjected to the previously described sample treatment procedure and fortified at different concentration levels (5, 10, 25, 100 and 250 μg/kg). Two samples for each concentration level were processed and injected in duplicate (*n* *=* 4). The peak area was considered as a response signal, being linearly dependent on the analyte concentration in the matrix sample. LODs and LOQs were determined as the minimum analyte concentration in the calibration curve yielding a signal-to-noise ratio equal to three and ten, respectively, based on the quantifier transition (Q).

The precision and recovery of the method was evaluated in terms of repeatability (intra-day precision) and intermediate precision (inter-day precision) at four concentration levels (L1, L2, L3 and L4) corresponding to 10, 25, 100 and 250 μg/kg, respectively. Repeatability was assessed over three samples for each concentration level and injected in triplicate (*n =* 9) on the same day under the same experimental conditions. Intermediate precision was evaluated with a similar procedure with a sample analyzed in triplicate for three consecutive days (*n =* 9). The recovery percentage (%R) was calculated by comparing the peak area of the target cyclic peptide cyanotoxins in a blank sample spiked before the sample treatment with those fortified on the extract from the end of the sample treatment process, just before injection. This comparative approach aimed to evaluate the efficiency and reliability of the sample extraction protocol regarding recovery rates.

ME was calculated over three samples for each concentration level, analyzed in triplicate (*n =* 9), according to Equation (1).

(1) ME (%) = [(A − B)/B] × 100

where A = the peak area of the analyte in the sample extract fortified after sample treatment and before the injection, and B = the peak area of the analyte in solvent standard solution.

### 2.5. Suspect Screening Analysis by HPLC-Q-Orbitrap-HRMS

The analysis was carried out with a Vanquish LC chromatograph (Thermo Fisher Scientific, Waltham, MA, USA) comprising a binary pump, a degasser, a temperature-controlled column compartment, and an autosampler. This system was coupled to a Q-Exactive™ hybrid quadrupole-Orbitrap high-resolution mass spectrometer (Thermo Fisher Scientific). Chromatographic separation was performed using the same column and mobile phases indicated in Section 2.3 but using a generic gradient for suspect and non-targeted analysis as follows: 0 min, 0% of eluent B; 2 min, 5% of eluent B; 19 min, 95% of eluent B; 24 min, 95% of eluent B; and 25.5 min, 5% of eluent B kept until 30 min for column equilibration. The flow rate was set at 0.3 mL/min and the column temperature at 40 C.

The HRMS operated in both positive and negative ESI modes, considering the following parameters. The heater temperature was 300 °C and the capillary temperature 300 °C. The auxiliary and sheath gas used was nitrogen (95%), the spray voltage was 4 kV, and the S-lens radio frequency level was 50 (arbitrary units). Full-scan mode was used for HRMS data acquisition of precursor ions in a mass-to-charge ratio (m/z) range of 70–1200 for both the ESI+ and ESI− modes, with a maximum injection time (IT) of 250 ms, an automatic gain control (AGC) target of 1 × 10^5^, and a resolution of 35,000 FWHM (m/z 200).

Data-dependent acquisition (DDA) was used to monitor fragment ions (MS/MS data) and was performed using the data-dependent dd-MS2 (Top5) mode within the *m/z* 80–12,000 range in both ESI+ and ESI− modes. This mode involved the higher-energy collisional dissociation (HCD) collision cell with normalized collision energies (NCEs) of 30 eV. The MS/MS data were acquired with a maximum IT of 125 ms, an isolation window of 5 *m/z*, an AGC target of 1 × 10^5^, a dynamic exclusion time of 10 s, and a resolution of 35,000 FWHM at *m/z* 200 for both polarities.

An inclusion list of predefined target precursor ions (m/z) was generated for the most common cyclic peptide cyanotoxins, including the target compounds anlaysed by HPLC-MS/MS, to broaden the screening scope. This approach ensured the selective fragmentation and subsequent identification of these compounds in complex sample matrices.

The UHPLC-Q-Orbitrap-HRMS data was acquired using the Xcalibur Sequence Setup software (Version 4.4, Thermo Fisher Scientific).

### 2.6. HRMS Workflow for Data Processing

A comprehensive workflow for the suspect screening analysis of cyanotoxins in spirulina- and klamath-based food supplements was developed using Compound Discoverer™ version 3.3 software (Thermo Fisher Scientific).

Initially, experimental data files (.raw) were imported into the software. The calibration curve containing the cyclic peptide cyanotoxins was categorized as “standard” to validate the workflow and enable the semi-quantification of potentially detected novel cyanotoxins not included in the target approach. In addition, other available standards of cyanotoxins that may be present in the target samples (e.g., SXT, ANA, CYN) were injected individually and categorized as “Identification Only” to use the fragmentation scans in the sample for component identification. Procedure blanks (i.e., solvent injections) categorized as “blanks” and quality control (QC) samples categorized as “quality control” (i.e., stardards mix solution) were included in the workflow for background signal removal and area refinement. Background filtering was carried out by setting the maximum allowed ratio of sample to blank at 5 for consideration as a blank feature.

Feature extraction in Compound Discoverer was performed within a retention time range of 1–25 min and an *m/z* range of 70–1200 for both ESI^+^ and ESI^−^ ionization modes. Additional extraction parameters included a mass tolerance of 5 ppm, minimum peak intensity of 1 × 10^5^, intensity tolerance of 30%, signal-to-noise (S/N) threshold of 3, intensity threshold of 0.1%, and retention time (RT) tolerance of 0.5 min. The preferred adducts included [M + H]^+^, [M + 2H]^2+^, [M + H-H_2_O]^+^, [M + Na]^+^, [M−H]^−^, [M + K]^+^, and [M + NH_4_]^+^.

Compound identification was performed by matching to LC-MS spectral libraries including ChemSpider, ChEBI, ChemBank, DrugBank, EPA Toxcast, FDA, MassBank, NIST, PubMed, Toxin (Toxin-Target Database), LIPID maps, PlantCyc, PubMed, Natural Products Atlas 2021_8, and mzCloud, with a mass tolerance of 5 ppm for precise annotations. An expected compound library was also built, including 23 cyanotoxins using the ChemSpider Search. The “Class Coverage” parameter was added to the workflow, providing a list of characteristic ions associated with the target analytes, in this case cyclic peptide cyanotoxins, particularly MCs.

The “Factor of Ionization Score” (FISh) option, available in Compound Discoverer, was used to evaluate the fragmentation coverage of the tentatively identified cyanotoxins. This score reflects the match between experimental fragment ions and predicted fragmentation patterns, providing a measure of confidence in the compound identification.

The identification of potential cyanotoxin compounds was performed according to the Schymanski identification levels [24].

Molecular networking was generated using the candidates identified after data refinement, using the Molecular Networking option in Compound Discoverer™. The optimization of the settings is described in Section 3.3.

## 3. Results and Discussion

### 3.1. Sample Treatment Evaluation

**Figure 1:**
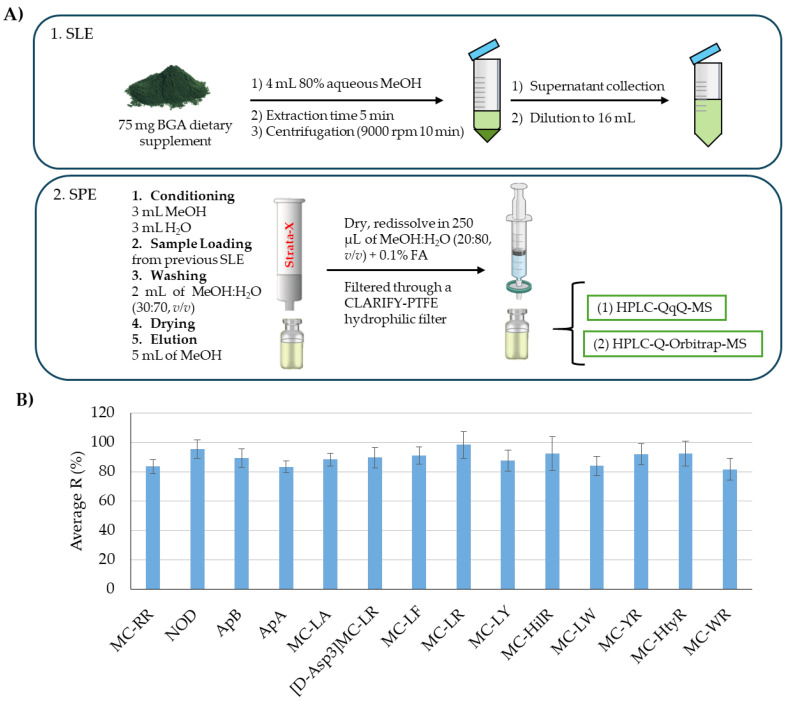
(**A**) Schematic sample treatment procedure for cyclic peptide cyanotoxin extraction and purification from BGA-based products; (**B**) bar plot illustrating the average recovery percentages of the 14 target compounds at 100 μg/kg. The error bars represent the associated standard error (*n =* 4).

Sample preparation for the extraction of cyanotoxins from BGA-based products typically involves solid–liquid extraction (SLE) using aqueous mixtures containing a high proportion of MeOH, acidified MeOH, or acidified water. The choice of solvent depends on the type of cyanotoxin being extracted. Following extraction, due to the complexity of these products, the samples are often purified using SPE. For lipophilic molecules like MCs, and NOD, hydrophobic SPE cartridges are commonly used, while hydrophilic ones such as BMAA are generally cleaned using cation exchange cartridges [25]. Although tandem SPE approaches have been proposed for the simultaneous extraction of multiple cyanotoxins, anabaenopeptins have not yet been effectively integrated into these SPE protocols for BGA-based food supplement analysis. Considering the literature available involving the extraction of cyclic peptide cyanotoxins in similar products, it was observed that some MCs (MC-RR and MC-LR) were successfully extracted by using Strata-X cartridges [16]. Thus, to test the applicability of this SPE procedure to a higher number of MCs and to expand the analysis to other cyclic peptide cyanotoxins, it was applied for the extraction of 11 MCs, anabaenopeptins (APA and APb), and NOD. The SPE procedure evaluated is represented in Figure 1A and described in Section 2.2.

Recovery was assessed by comparing the peak areas of target compounds in blank samples spiked before the extraction procedure with those fortified at the corresponding concentration after extraction immediately prior to LC-MS/MS analysis. The recovery assays were conducted in two experimental replicates and injected in duplicate (*n* = 4).

As observed in Figure 1B, for all target compounds, the recovery was above 80%, so no further optimization was needed. Therefore, it was confirmed that the previously optimized sample treatment [20] can be also applied to a higher number of related compounds such as MC-YR, MC-WR, MC-LA, MC-LY, MC-LW, MC-LF, MC-HliR, MC-HtyR, and [DAsp3]-MC-LR, as well as for the ApA and ApB, which were first tested under such conditions. These results also suggest that this protocol could be applied to other similar compounds not included in this study, particularly MCs.

### 3.2. Targeted UHPLC-MS/MS Method

The most intense precursor and product ion MRM transitions as well as the optimal fragmentor voltages and collision energies were obtained automatically by using the Optimizer optimization tool (version 10.1) in Agilent Mass Hunter software. Protonated [M + H]^+^, doubly protonated [M + 2H]^2+^, and deprotonated [M − H]^−^ molecular ions were monitored as precursor ions depending on the cylic peptide. For each compound, two characteristic fragment ions were selected and monitored to ensure reliable identification and quantification (Table 1). The ‘Dynamic MRM’ (dMRM) acquisition mode available in the Agilent MassHunter Workstation was evaluated and compared with the traditional MRM mode to assess improvements in sensitivity, selectivity, and data acquisition efficiency. Thus, compound-specific retention time windows were applied to monitor each analyte based on its expected elution time. This approach allowed for longer dwell times per transition, resulting in increased signal intensity and reduced background noise. Consequently, the use of dMRM significantly enhanced the overall sensitivity of the method.

The chromatographic separation was based on a previous commercial application note involving the use of an Acquity UHPLC BEH C18 column for the separation of diverse cyclic peptide cyanotoxins [26]. Column selection was primarily guided by the physicochemical properties of the target analytes, as cyclic peptide are typically amenable to reversed-phase separation [27]. This column provided good performance for these target compounds, particularly in terms of peak shape, resolution, and reduced analysis time.

### 3.3. UHPLC-MS/MS Method Characterization

The characterization of the method, including linear range, sensitivity, LODs and LOQs, extraction efficiency, precision (i.e., repeatability and intermediate precision) and ME, was performed as described in Section 2.4.

Matrix-matched calibration curves were established in the representative spirulina samples, which were submitted to the sample treatment procedure and fortified at different concentration levels (5, 10, 25, 100 and 250 μg/kg). Statistical parameters calculated via least-squares regression, along with the performance characteristics of the method, are detailed in Table 2.

Satisfactory linearity was achieved across the linear range with determination coefficients of R^2^ > 0.9900 in all cases. The LOQs were in the low μg/kg levels ranging from 5 to 20 μg/kg depending on the analyte. Compared to previous HPLC-MS/MS methods for assessing these cyanotoxins in BGA-derived dietary supplements, the proposed method achieved notably lower LOQs [2]. This improvement is attributed to the study’s exclusive focus on the cyclic peptide group, which allowed for the analysis of a higher number of these compounds while concurrently enhancing the method’s sensitivity.

The results of the precision study, expressed as relative standard deviation (% RSD) of the peak areas, are also shown in Table 2. All compounds showed satisfactory precision, with % RSDs lower than 20% across the tested concentration levels.

In order to assess the trueness of the proposed analytical method, recovery experiments were carried out using four different concentration levels of cyanotoxins (L1, L2, L3 and L4 corresponding to 10, 25, 100 and 250 μg/kg, respectively). This approach aimed to evaluate the efficiency and reliability of the sample extraction protocol regarding recovery rates. The recoveries obtained at each concentration level are shown in Table 3. In all cases, the recovery was above 70% with RSDs below 20%, even at the lowest levels, indicating acceptable variability of the recovery measurements across the different levels and analytes.

In this study, ME refers to the influence of components derived from BGA-based food supplements other than the target analytes on the analytical response. ME was assessed at four concentration levels (L1, L2, L3, and L4) corresponding to 10, 25, 100, and 250 μg/kg, respectively. For those analytes whose LOQ was higher than 10 μg/kg (i.e., [D-Asp3]MC-LR, MC-HilR and MC-WR), ME was evaluated at L2, L3, and L4. This assessment involved comparing the analyte response obtained from a blank extract of BGA-based dietary supplement fortified with the analytes post-sample treatment, with the response from a solvent standard solution of the analytes at identical concentration levels. The average ME observed for each analyte is shown in Table 3.

An ME value of 0% indicates the absence of ME, whereas a value higher or lower than 0% represents signal enhancement or suppression, respectively. Several cyclic peptides, such as MC-RR, NOD, ApA, MC-LY, MC-HilR, MC-LW, and HtyR, exhibited significant matrix-induced signal suppression, with ME values exceeding −20%. Conversely, [D-Asp3]MC-LR, MC-LR, MC-YR, and MC-WR demonstrated minimal influence from the matrix, with ME values below 10%.

### 3.4. Workflow for Suspect Screening Analysis

A comprehensive workflow was developed, utilizing the tools described in Section 2, for the suspect screening analysis of cyclic peptides in spirulina- and klamath-based food supplements.

First, feature extraction was carried out according to the Compound Discoverer criteria described in Section 2.5. The order of preference for assigning the possible identity of the analyte was established as follows: mzCloud, Mass List Search, ChemSpider Search, and finally, Predicted Composition.

Identification was based on common MS1 libraries as from the Mass List and ChemSpider nodes, ensuring that the calculated mass error aligned with the proposed structures derived from the previous nodes. An in-house built library was also added to the Mass List containing up to 50 formula and molecular *m/z* of potentially present cyanotoxins. Recent articles based on the molecular identification of novel and less common cyclic peptide cyanotoxins were very useful [21,28]. Additionally, a “Class Coverage” parameter was incorporated into the workflow to further refine candidates. This step involved entering a list of eight characteristic fragment ions associated with the target compounds (Table S3). Specifically, known fragmentation patterns of MCs—particularly the Adda fragment ions—were considered, as described in the literature [21]. The Adda moiety (3-amino-9-methoxy-2,6,8-trimethyl-10-phenyldeca-4,6-dienoic acid) is a unique and conserved structural component of most MC variants and nodularins, and its presence is considered a hallmark in the MS/MS spectra of these compounds. Variations include 9-O-desmethylAdda (DMAdda) and 9-O-acetylDMAdda (ADMAdda). In addition, characteristic fragment ions such as *m/z* 135 (protonated Adda), ~213 (Adda–Glu fragment), and ~375 (Adda-derived fragment), which correspond to cleavage products of the Adda side chain, serve as reliable indicators for the presence of MCs. Some specific amino acids present in their structure were also considered (Figure S1). Incorporating these diagnostic fragments into the “Class Coverage” node of the workflow significantly improved the accuracy of suspect screening by filtering out non-relevant signals and confirming the presence of MC-like structures, even in the absence of complete library matches. This strategy enhanced the confidence in the annotation of suspect cyanotoxins, including potentially novel or modified variants that retain the Adda structural motif. Although the exact *m/z* values can vary slightly depending on the specific MC congener, instrument type, and collision energy settings, the *m/z* 135 is widely recognized as the core diagnostic ion representing the Adda moiety in tandem MS spectra.

Filters were applied throughout the workflow to reduce the number of candidate analytes, with parameters specifically adapted to the requirements of this study. The signals present in both blank and sample measurements were removed to eliminate background noise. Only features with mass errors between −5 and 5 ppm were retained. A chromatographic peak rating greater than 4 was required, ensuring the retention of real and well-defined signals. Retention time was restricted to the range of 1 to 20 min.

This workflow was validated by identifying cyclic peptide cyanotoxins for which analytical standards were available, thereby confirming the reliability of the procedure. A schematic representation of the Compound Discoverer workflow nodes is provided in the Supplementary Materials (Figure S2). After data processing using this workflow, a filter was created to apply the Mass List and Class Coverage previously described. Additional restrictions based on area, background, peak rating, and mzCloud results were implemented to further reduce the number of identified compounds (Figure S3). Finally, manual review and annotation were performed once the number of candidate compounds was reduced.

Finally, the “Molecular Networks” node was added to evaluate the structural relationships between cyanotoxins and potentially related analytes. Thus, by clustering compounds with similar fragmentation profiles, this approach facilitates the identification of unknown or structurally related compounds through their association with known cyanotoxins.

### 3.5. Comprehensive Analysis of Comercial BGA Food Supplements

After the proposed sample treatment procedure was applied to the commercial samples, the extracts were analyzed using both UHPLC-MS/MS and UHPLC-Q-Orbitrap-MS methods.

**Figure 2:**
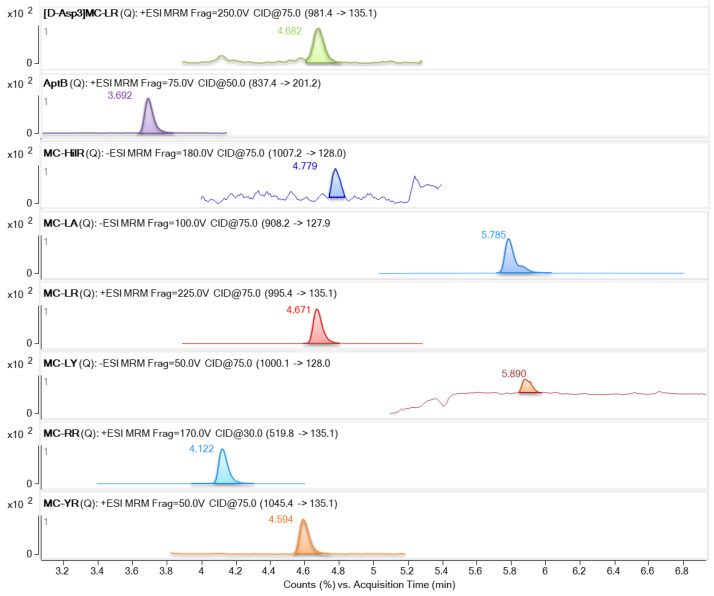
Extracted ion chromatograms of the cyclic peptide cyanotoxins detected in a klamath sample (FS-8) by UHPLC-MS/MS. Detected target compounds are highlithed in bold, along with their corresponding transitions.

In the case of the targeted UHPLC-MS/MS method, several samples were found to be contaminated with some of the target cyclic peptides, as shown in Table 4. Sample FS-8, composed of pure klamath (Aphanizomenon flos-aquae), exhibited the highest level of contamination, with the co-occurrence of nearly all target cyclic peptide cyanotoxins at relatively high concentration levels, particularly for several MCs, including MC-RR, MC-LA, MC-LR, and MC-YR (Figure 2). Notably, MC-RR was detected at a concentration of 3262.3 μg/kg. These MCs have also been reported together in previous studies on klamath-based products at high concentrations, especially MC-LR and MC-LA [17,29]. On the other hand, MC-LF was found in three samples mainly composed of spirulina (Arthrospira platensis); however, no other MC congeners were found in these samples.

Subsequently, the same sample extracts were injected into the UHPLC-Q-Orbitrap-MS system in order to identify additional cyclic peptide cyanotoxins by a suspect screening analysis using the workflow for suspect screening described in Section 3.3. A calibration curve containing the target cyclic peptides included in the targeted method was also injected to verify the reliability of the injection and to ensure a proper operation of the workflow during data treatment. The contamination detected in the previously assigned positive samples was corroborated by the identification of the corresponding compound using this approach.

In some cases, the use of the FISh scoring tool was helpful for the accurate identification of MCs. For instance, MC-HilR was initially identified at two different retention times; however, one proposed assignment yielded a FISh score of 63, while in the other case, scoring using this tool was not feasible (Figure S4). Although both proposed identifications presented matches with ChemSpider and mzCloud, in the second case, the Class Coverage (fragment coverage of individual compound class) was not available, resulting in a FISh score of 0. This fact highlights the importance of adding the Class Coverage filter and using the FISh tool for comparing fragmentation when multiple assigments are proposed for the same compound and standards are not available. In addition, the FISh score proved to be useful when similar data was obtained from ChemSpider, mzCloud, and Class Coverage for two different assignments, as was the case for MC-YR, where the Class Coverage was 50 in both cases. Small differences in FISh coverage and the signal intensity of major fragments can help to achieve the right identification under these conditions. In both cases, the identification of MC-HilR and MC-YR was confirmed by using analytical standards for retention time comparison.

This approach was also used to investigate other MC congeners, focusing specifically on FS-8 as it presented the highest concentration of MCs. The hypotesis was that, due to the high concentration of several MCs, such as MC-RR and MC-LR, it was possible to observe variants of such MCs. To explore this, the main variants of these congeners were first investigated in the literature, and then potential identifications with high Class Coverage (above 30) were compared with the available structural information. [D-Asp^3^]MC-RR, [M + H]^+^ = 1025.523 *m/z* and (L-NMe-Ala7)MC-LR ([M + H]^+^ = 997.621 *m/z*) were putatively identified with Schymanski identification level 3, while [D-Asp^3^, D-Asp^6^]MC-RR, [M + H]^+^ = 1011.508 *m/z* was tentatively identified with level 2. In addition, MC-WL was tentatively identified (level 3) based on its *m/z* and fragmentation pattern, which showed several characteristic fragments related to its amino acid composition (e.g., Ala = 71.0371, Pro = 97.0528). However, the main Adda fragments were not observed, which limits the confidence in its structural elucidation. Therefore, reference standards or complementary analytical techniques would be required for confirmation, justifying its classification at level 3 within the Schymanski framework.

Once the compounds were identified or tentatively identified after data refinement, molecular networking was performed using that option in Compound Discoverer. To maximize the potential of molecular networking, the Class Coverage filter was removed, expanding the analyte pool to 1500 compounds and generating a comprehensive MS2-based fingerprint. This revealed the samples’ chemical complexity and highlighted clusters likely representing compound families. To properly observe the cylic peptide cluster, the following parameters were adjusted. We used score and coverage thresholds of 25 in both cases, at least 20 matched fragments, a maximum of 10 node links, and a maximum of cluster size of 100. These settings helped to focus the network on strong and reliable relationships between compounds. As shown in Figure S5, a well-defined cluster was obtained for the identified cyclic peptide, offering a good strategy for identifying additional related cyanotoxins. However, since no interconnections with other clusters were observed, the discovery of other related compounds was limited. If the objective was to identify new compounds, reducing the required number of matched fragments could have allowed interconnections with other identified compounds in the network. This strategy may be particularly valuable when analyzing cyanobacterial blooms, where the high concentrations of these compounds can facilitate the discovery of less-studied cyanotoxins or aid in the elucidation of unknown variants.

## 4. Conclusions

An integrated targeted and suspect screening workflow using UHPLC-MS/MS and HRMS was successfully applied for the comprehensive profiling of cyclic peptides in spirulina- and klamath-based food supplements.

The sample treatment based on a previous SLE and SPE clean-up demonstrated high recovery rates and low matrix effects across a broad range of cyanotoxins, confirming its suitability for these complex matrices. The targeted analysis revealed the presence of multiple MC congeners and ApA in several samples, with the highest contamination levels observed in a klamath-based supplement where the concentrations exceeded 3000 μg/kg for MC-RR and substantial levels were also found for other MCs. The suspect screening expanded detection beyond the scope of the target method with the putative identification of other variants such as [D-Asp^3^]MC-RR, and [D-Asp^3^, D-Asp^6^]MC-RR in such samples.

The incorporation of Class Coverage filtering and FISh scoring within the suspect screening workflow enhanced the confidence in compound annotation, particularly under conditions where analytical standards were unavailable. Moreover, molecular networking proved to be useful for visualizing cyanotoxin clustering, facilitating the interpretation of complex matrices. However, the applicability of this approach should be further investigated in BGA-producing environments to evaluate its potential for discovering additional cyanotoxins.

Overall, the findings of this study highlight the need for systematic monitoring and regulatory frameworks to control cyanotoxin contamination in BGA-based supplements in order to ensure consumer safety. The combination of advanced analytical strategies offers a valuable approach to the identification of known and emerging cyanotoxins in dietary products, supporting quality control while responding to the increasing need to investigate these underexplored matrices.

## Figures and Tables

**Table 1:** Dynamic multiple reaction monitoring (dMRM) parameters for the determination of cyclic peptide cyanotoxins by UHPLC-ESI-MS/MS.

Cyclic Peptide Cyanotoxins	Molecular Ion	Retention Time (min)	Precursor Ion (*m/z*)	Fragmentor Voltage (V)	Product Ion (m/z)	Collision Energy (eV)
NOD	[M + H]^+^	4.5	825.4	270	135.1 (Q) ^a^ 70.2 (q) ^b^	75 85
MC-LR	[M + H]^+^	4.7	995.4	225	135.1 (Q) 127.0 (q)	75 100
[D-Asp3] MC-LR	[M + H]^+^	4.7	981.4	250	135.1 (Q) 86.3 (q)	75 75
MC-RR	[M + 2H]^2+^	4.3	519.8	170	135.1 (Q) 70.2 (q)	30 100
MC-HilR	[M − H]^−^	4.8	1007.2	180	128.0 (Q) 279.1 (q)	75 75
MC-HtyR	[M + H]^+^	4.7	1059.4	250	135.1 (Q) 107.1 (q)	100 125
ApA	[M − H]^−^	4.4	842.1	75	661.1(Q) 824.1 (q)	30 20
ApB	[M + H]^+^	3.7	837.4	75	201.2 (Q) 70.2 (q)	50 100
MC-LA	[M − H]^−^	5.8	908.2	100	127.9 (Q) 282.9 (q)	75 50
MC-LF	[M − H]^−^	6.5	984.1	75	128.0 (Q) 109.9 (q)	75 75
MC-LW	[M − H]^−^	6.3	1023.2	100	127.7 (Q) 265.0 (q)	75 75
MC-WR	[M + H]^+^	4.9	1068.4	100	135.1 (Q) 70.2 (q)	75 160
MC-YR	[M + H]^+^	4.6	1045.4	50	135.1 (Q) 70.1 (q)	75 160
MC-LY	[M − H]^−^	5.9	1000.1	50	128 (Q) 264.9 (q)	75 75

^a^ Q: Product ion selected for quantification purposes; ^b^ q: Product ion selected for confirmation purposes.

**Table 2:** Performance characteristics for the proposed SPE-UHPLC-MS/MS method in BGA-based food supplements. Precision is expressed as relative standard deviation (RSD, %) at L1, L2, L3, and L4 which correspond to 10, 25, 100, and 250 μg/kg, respectively.

Analyte ^a^	Linear Range (μg/kg)	LOD (μg/kg)	LOQ (μg/kg)	R^2^	Intra-Day Precision (RSD, %) (*n =* 9)				Inter-Day Precision (RSD, %) (*n =* 9)			
					L1	L2	L3	L4	L1	L2	L3	L4
MC-RR	10–500	3.0	10.0	0.9920	16.5	12.4	2.1	11.2	15.2	5.8	8.4	12.5
NOD	5–500	1.5	5.0	0.9909	12.7	16.2	2.7	11.1	5.6	10.1	2.6	10.4
ApB	10–500	3.0	10.0	0.9900	6.8	2.4	1.4	11.2	4.5	8.1	9.9	12.3
ApA	10–500	3.0	10.0	0.9931	18.9	16.7	8.7	10.2	10.0	13.1	7.6	6.2
MC-LA	10–500	3.0	10.0	0.9924	13.4	16.4	2.8	9.0	1.8	11.0	5.0	7.3
[D-Asp3] MC-LR	10–500	5.2	10	0.9918	12.3	17.0	4.9	4.7	16.1	13.1	2.8	6.1
MC-LF	10–500	3.0	10.0	0.9948	12.8	19.0	6.9	7.3	14.0	14.1	5.7	4.9
MC-LR	10–500	3.0	10.0	0.9942	19.4	19.0	5.6	8.4	11.2	11.6	6.0	7.8
MC-LY	10–500	3.0	10.0	0.9964	16.8	19.8	3.4	11.2	18.9	11.3	2.9	9.4
MC-HilR	10–500	5.2	10	0.9959	18.6	15.1	7.3	8.7	17.8	13.0	7.4	9.8
MC-LW	10–500	3.0	10.0	0.9907	15.8	19.5	7.9	11.6	15.1	10.9	6.7	6.8
MC-YR	10–500	3.0	10.0	0.9904	12.1	15.5	7.4	12.1	15.6	14.1	7.7	10.6
MC-HtyR	10–500	3.0	10.0	0.9954	19.4	12.4	5.9	10.3	14.2	16.8	5.4	10.1
MC-WR	10–500	6.0	20.0	0.9924	18.2	10.8	7.1	11.8	17.4	12.7	10.3	11.4

^a^ Abbreviations: Ap: Anabaenopeptin; MC: Microcystin; NOD: Nodularin.

**Table 3:** Recovery (%) with the corresponding RSD (%) and the ME (%) for the proposed SPE-UHPLC-MS/MS) method in BGA-based dietary supplements. L1, L2, L3, and L4 correspond to 10, 25, 100, and 250 μg/kg, respectively.

Cyclip Peptide Cyanotoxin ^a^	Recovery (*n = 4*), % (RSD,%)				ME, %
	L1	L2	L3	L4	Mean (*n = 4*)
MC-RR	74.7 (7.1)	75.3 (16.2)	83.4 (4.0)	78.7 (7.0)	−37.4
NOD	72.4 (8.6)	75.4 (15.5)	95.3 (4.4)	79.3 (10.5)	−23.5
ApB	77.9 (4.0)	78.1 (3.3)	89.3 (4.1)	82.1 (10.3)	9.0
ApA	81.9 (12.3)	70.3 (10.5)	83.3 (10.6)	86.1 (5.5)	−33.7
MC-LA	76.3 (14.7)	72.7 (16.4)	88.2 (3.8)	85.5 (6.1)	−18.4
[D-Asp3]MC-LR	76.7 (14.1)	71.5 (9.1)	89.6 (6.6)	75.0 (8.4)	15.5
MC-LF	83.6 (6.0)	72.5 (9.0)	91.1 (8.2)	89.2 (4.7)	−11.5
MC-LR	70.9 (20.1)	80.0 (16.0)	98.2 (4.4)	84.7 (10.9)	6.0
MC-LY	74.4 (17.6)	72.3 (14.8)	87.6 (4.3)	80.9 (14.9)	−34.8
MC-HilR	77.7 (21.1)	82.6 (17.7)	92.3 (13.4)	92.5 (7.0)	−39.1
MC-LW	76.9 (14.5)	72.6 (20.0)	83.9 (13.7)	87.8 (1.8)	−38.0
MC-YR	79.7 (20.3)	75.0 (8.3)	91.7 (4.6)	84.5 (13.0)	6.2
MC-HtyR	73.2 (19.7)	72.3 (16.7)	92.3 (11.4)	92.8 (12.6)	25.7
MC-WR	72.7 (16.7)	73.5 (40.5)	81.6 (14.6)	69.4 (19.0)	18.3

^a^ Abbreviations: Ap: Anabaenopeptin; MC: Microcystin; NOD: Nodularin.

**Table 4:** Mean concentrations of positive samples (μg/kg). Not detected (<LOD); detected but not quantified (<LOQ).

Target Cyclic Peptides	FS-1	FS-2	FS-3	FS-4	FS-5	FS-6	FS-7	FS-8
MC-RR	<LOD	<LOD	<LOD	<LOD	<LOD	<LOD	<LOD	3262.3
NOD	<LOD	<LOD	<LOD	<LOD	<LOD	<LOD	<LOD	<LOD
AptB	<LOQ	<LOQ	<LOQ	<LOQ	<LOQ	<LOQ	<LOQ	87.8
AptA	<LOD	<LOD	<LOD	<LOD	<LOD	<LOD	<LOD	<LOD
MC-LA	<LOD	<LOD	<LOD	<LOD	<LOD	<LOD	<LOD	566.3
[D-Asp3]MC-LR	<LOD	<LOD	<LOD	<LOD	<LOD	<LOD	<LOD	92.9
MC-LF	<LOQ	19.4	<LOQ	<LOD	<LOD	12.0	<LOD	<LOD
MC-LR	<LOD	<LOQ	<LOQ	<LOD	<LOD	<LOD	<LOD	1738.2
MC-LY	<LOD	<LOD	<LOD	<LOD	<LOD	<LOD	<LOD	<LOQ
MC-HilR	<LOD	<LOD	<LOD	<LOD	<LOD	<LOD	<LOD	21.5
MC-LW	<LOD	<LOD	<LOD	<LOD	<LOD	<LOD	<LOD	<LOD
MC-YR	<LOD	<LOD	<LOD	<LOD	<LOD	<LOD	<LOD	941.1
MC-HtyR	<LOD	<LOD	<LOD	<LOD	<LOD	<LOD	<LOD	<LOD
MC-WR	<LOD	<LOD	<LOD	<LOD	<LOD	<LOD	<LOD	61.0

## Data Availability

The original contributions presented in this study are included in the article/Supplementary Materials. Further inquiries can be directed to the corresponding author.

## Supplementary Materials

The following supporting information can be downloaded at: https://www.mdpi.com/article/10.3390/foods14172969/s1, Table S1. Structural information and main characteristics of the target cyclic peptide cyanotoxins. Table S2. Detailed information on the investigated samples based on spirulina and Klamath. Table S3. Common fragments observed during microcystin fragmentation. Figure S1. General structure of microcystins with amino acid common positions and residue masses. Other variable amino acids in positions 2 and 4 (X, Z). Figure S2. Schematic representation of the Compound Discoverer workflow nodes for suspect screening analysis. Figure S3. Filter applied in Compound Discover for data processing. Figure S4. FISh score for potential identifications of MC-HilR in the suspect screening by UHPLC-Q-Orbitrap-MS. Figure S5. Molecular networking of the target food supplements highlighting the cyanotoxin cluster and its differentiation among the identified compounds.
