# Supplementary material for: Application of Targeted and Suspect Screening Workflows for Cyclic Peptide Cyanotoxin Profiling in Spirulina- and Klamath-Based Food Supplements
Source: Foods. 2025 Aug 26;14(17):2969. doi: 10.3390/foods14172969 (PMC12428515; doi:10.3390/foods14172969)
Supplement: Supplementary file 1 [file foods-14-02969-s001.zip › foods-3805735-supplementary.pdf]

SUPPLEMENTARY MATERIALS

Application of Targeted and Suspect Screening Workflows for Cyclic Peptide Cyanotoxins Profiling in Spirulina- and Klamath-Based Food Supplements

Laura Carbonell-Rozas[1,2*], M. Mar Aparicio-Muriana[1], Roberto Romero-González[2], Antonia Garrido Frenich[2], Ana M. García-Campaña[1], Monsalud del Olmo-Iruela[1]

[1] Department of Analytical Chemistry, University of Granada, Av. Fuente Nueva s/n, 18071 Granada, Spain.
[2] Department of Chemistry and Physics, Research Centre for Mediterranean Intensive Agrosystems and Agrifood Biotechnology (CIAIMBITAL), Agrifood Campus of International Excellence (ceiA3), University of Almeria, E-04120 Almeria, Spain.

*Correspondence: rozas@ugr.es

Table S1. Structural information and main characteristics of the target cyclic peptide cyanotoxins.

Name	Formula	Molecular weight	Purity	Chemical structure
Microcystin-Leucine-Arginine (MC-LR)	$C_{49}H_{74}N_{10}O_{12}$	994.5488	≥99%	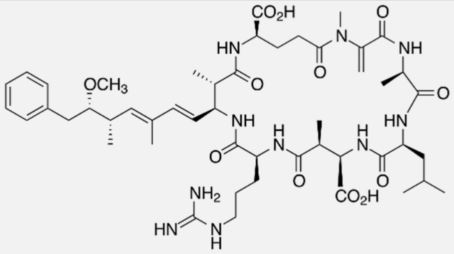
Microcystin-Arginine-Arginine (MC-RR)	$C_{49}H_{75}N_{13}O_{12}$	1037.5658	≥99%	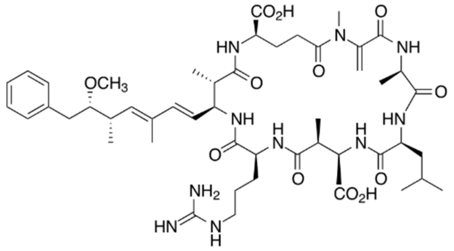
Microcystin-Tryptophan-Arginine (MC-WR)	$C_{54}H_{73}N_{11}O_{12}$	1067.5440	≥99%	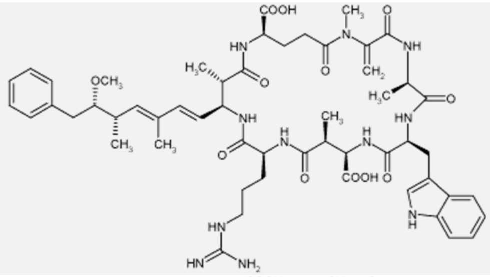
Microcystin-Homoisoleucine-Arginine (MC-HilR)	$C_{50}H_{76}N_{10}O_{12}$	1008.5644	≥99%	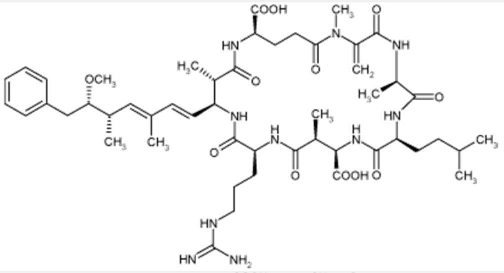
Microcystin-Homotyrosine-Arginine (MC-HtyR)	$C_{53}H_{74}N_{10}O_{13}$	1058.5437	≥99%	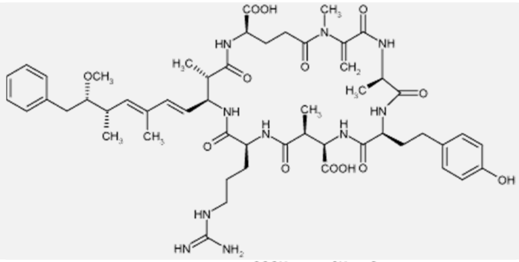
Microcystin-Tyrosine-Arginine (MC-YR)	$C_{52}H_{72}N_{10}O_{13}$	1044.5280	≥99%	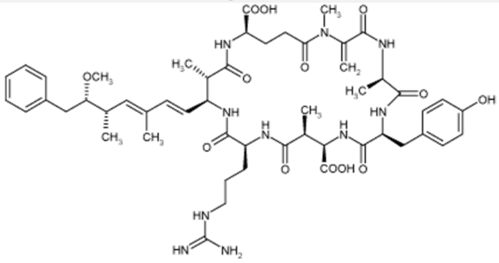

Microcystin-Leucine-Tryptophan (MC-LW)	$C_{54}H_{72}N_8O_{12}$	1024.5270	≥99%	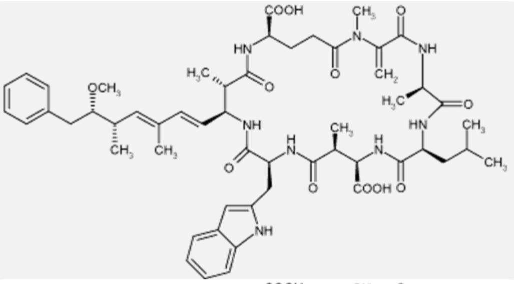
Microcystin-Leucine-Tyrosine (MC-LY)	$C_{52}H_{71}N_7O_{13}$	1001.5110	≥99%	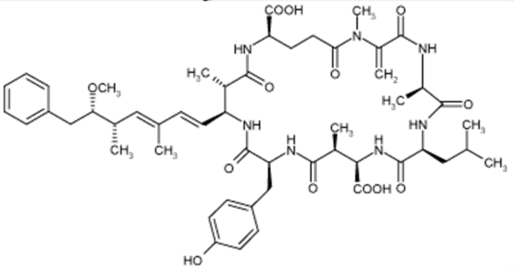
[D-Asp3]Microcystin-Leucine-Arginine ([D-Asp3]-MC-LR)	$C_{48}H_{72}N_{10}O_{12}$	980.5331	≥99%	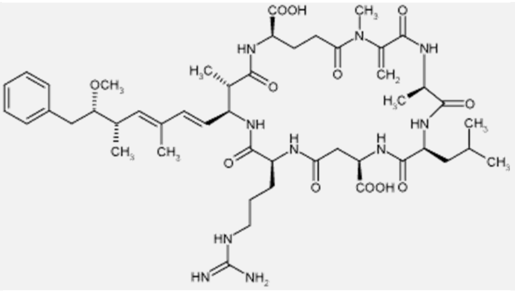
Microcystin-Leucine-Phenilalanine (MC-LF)	$C_{52}H_{71}N_7O_{12}$	985.5161	≥99%	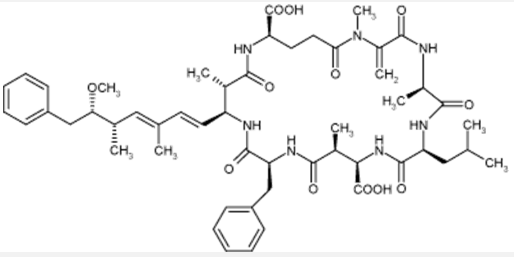
Microcystin-Leucine-Alanine (MC-LA)	$C_{46}H_{67}N_7O_{12}$	909.4848	≥99%	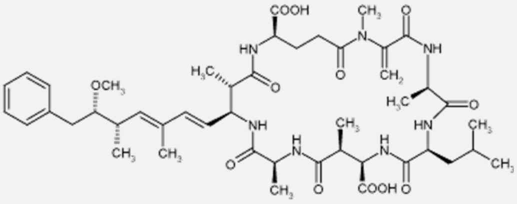
Anabaenopeptin A (APa)	$C_{44}H_{57}N_7O_{10}$	843.4167	≥95%	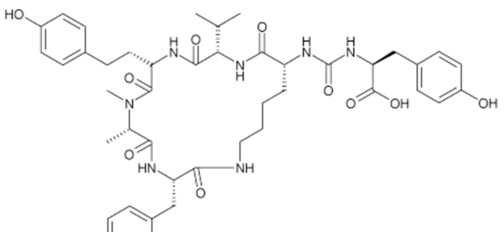
Anabaenopeptin B (APb)	$C_{41}H_{60}N_{10}O_9$	836.4545	≥95%	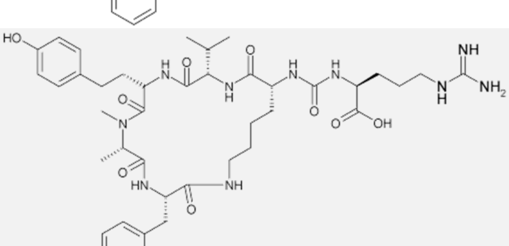

Nodularin (NOD)	$C_{41}H_{60}N_8O_{10}$	824.4432	≥95%	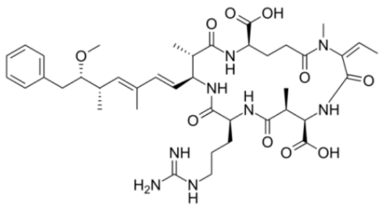

Table S2. Detailed information on the investigated samples based on spirulina and klamath.

Sample code	Supplier	Form	Composition	Daily dose
**FS-1**	Local Store	Powder	Pure Spirulina (*Arthrospira platensis*)	Not available
**FS-2**	Local store	Powder	Pure spirulina (*Arthrospira platensis*)	6 g product, i.e, 6000 mg spirulina
**FS-3**	Online	Tablet	Spirulina (*Spirulina platensis* Geitler), dioxide de silício	6 capsules, i.e, 2562 mg spirulina
**FS-4**	Local Store	Capsule	Spirulina (*Arthrospira platensis*), hydroxypropyl methylcellulose	6 capsules, i.e, 2160 mg spirulina
**FS-5**	Online	Tablet	Spirulina (*Arthrospira platensis*), chlorella (*Chlorella vulgaris),* ferrous fumarate, folic acid, vitamin B12	3 capsules, i.e, 750 mg spirulina, 750 mg chlorella
**FS-6**	Online	Capsule	Spirulina (*Arthrospira platensis* Gomont), hydroxypropyl methylcellulose, chlorella (*Chlorella vulgaris* Beijerinck), fucus (*Fucus vesiculosus* L.), vitamin C, magnesium stearate	6 capsules, i.e, 891 mg spirulina, 445.5 mg chlorella, 445.5 mg fucus
**FS-7**	Online	Tablet	Klamath (*Aphanizomenon flos-aquae*), spirulina (*Spirulina platensis* Geitler), microcrystalline cellulose, silicon dioxide	3 capsules, i.e, 600 mg klamath, 600 mg spirulina
**FS-8**	Local store	Powder	Pure Klamath (*Aphanizomenon flos-aquae*)	Not available

Table S3. Common fragments observed during microcystin fragmentation.

**Fragment ID**	***m/z***
Adda	313.2042
Aromatic Adda fragment	135.0446
ADMAdda	341.1991
DMAdda	299.1885
Glu	129.0426
Arg	156.1011
Adda-Glu	213.0872
Mdha-Ala	142.0737
D-Glu+Mdha	213.0870
Mdha+D-Ala	127.0866
MeAsp	129.0426
Ala	71.0371
Mdhb	97.0528

Adda = (2S,3S,8S,9S)-3-amino-9-methoxy-2,6,8-trimethyl-10-phenyldeca-4,6-dienoic acid; Mdha = N-methyldehydroalanine, Dha = dehydroalanine; Dhb = dehydrobutyric acid; Mdhb = 2-(methylamino)-2(Z)-dehydrobutyric acid; DMAdda= 9-O-desmethylAdda and ADMAdda=9-O-acetylDMAdda

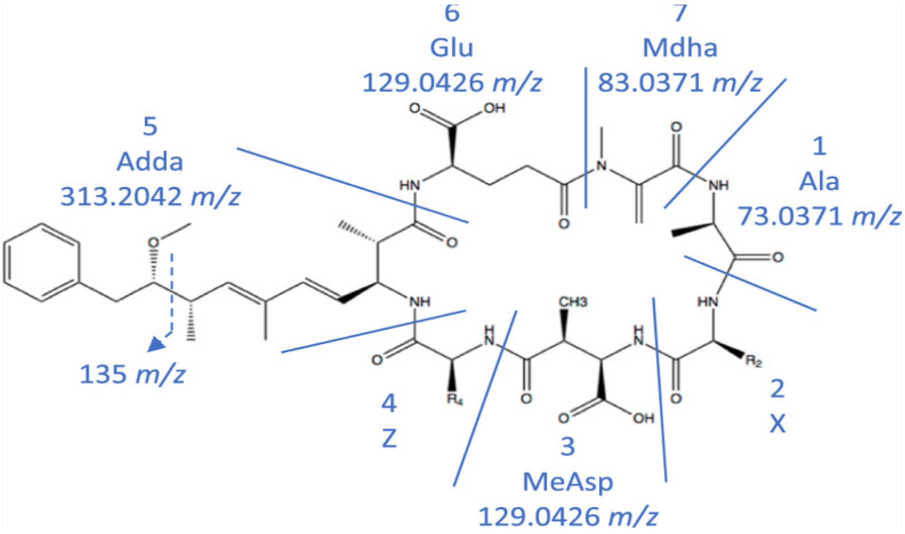

Figure S1. General structure of microcystins with amino acid common positions and residue masses. Other variable amino acids in positions 2 and 4 (X, Z).

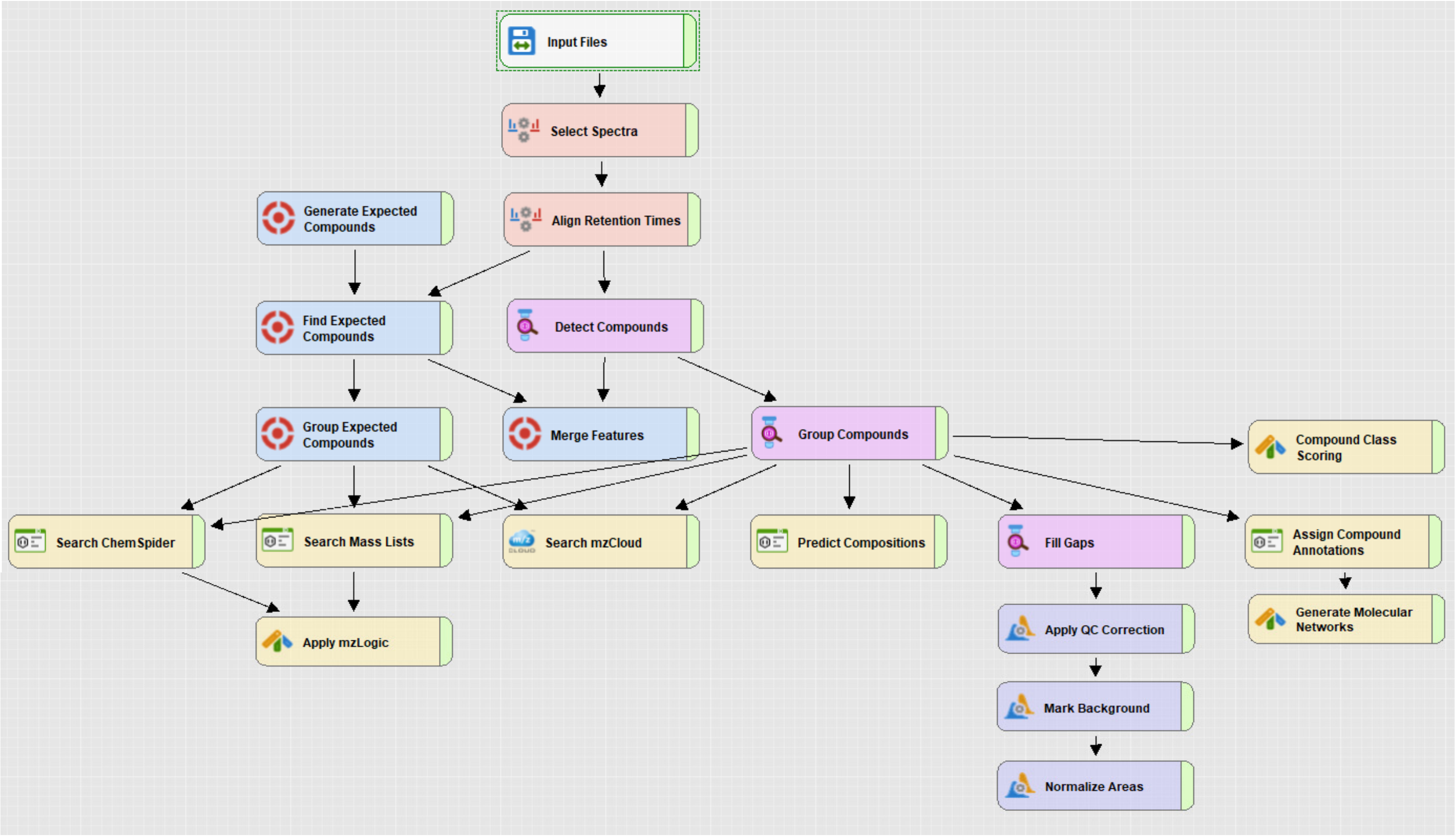

Figure S2. Schematic representation of the Compound Discoverer workflow nodes for suspect screening analysis.

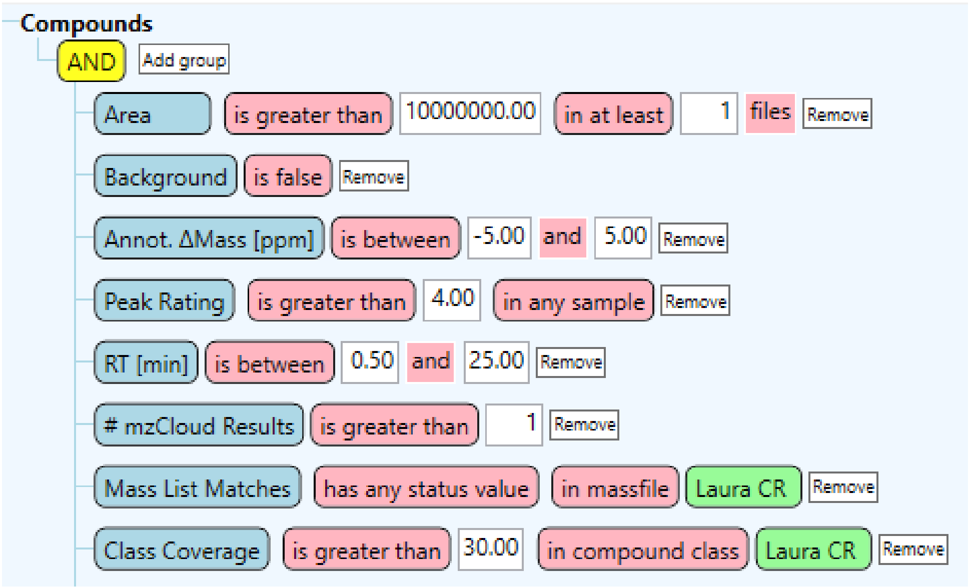

Figure S3. Filter applied in Compound Discover for data processing.

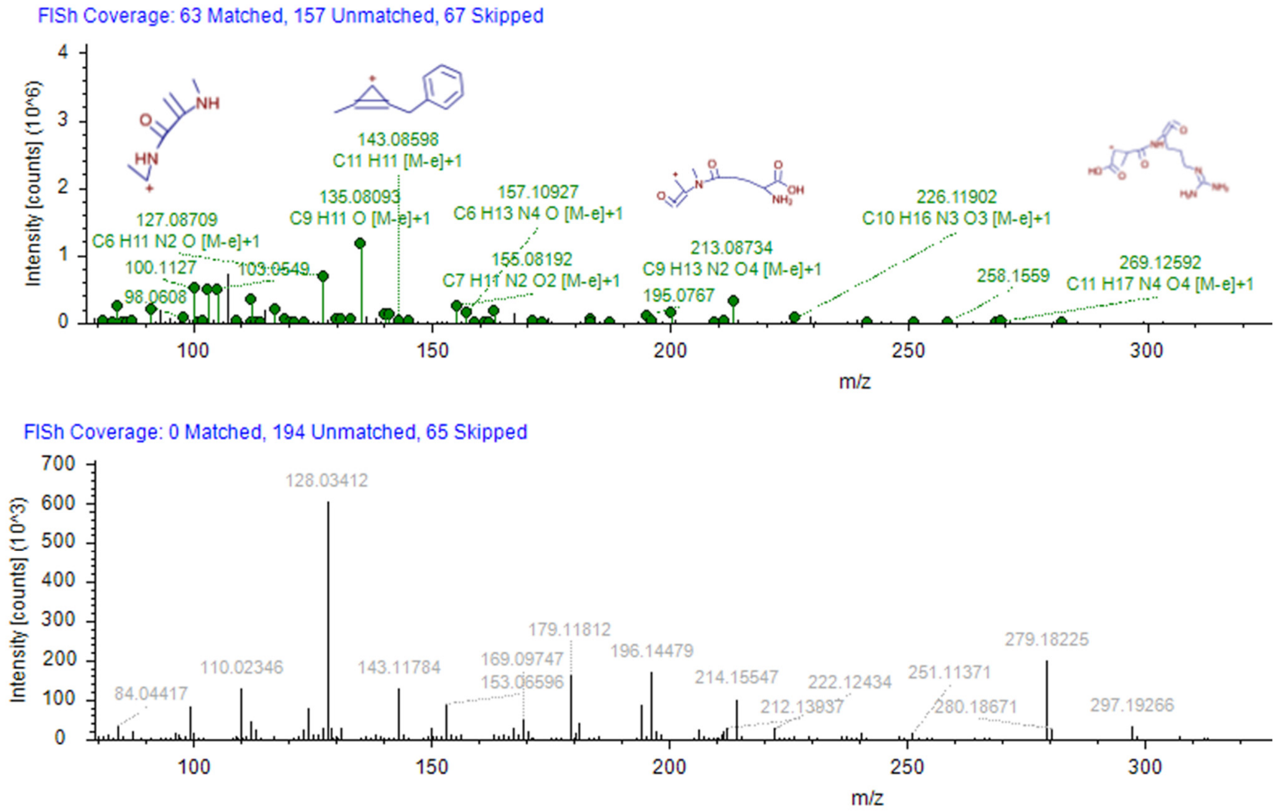

Figure S4. FISh score for potential identifications of MC-HilR in the suspect screening by UHPLC-Q-Orbitrap-MS.

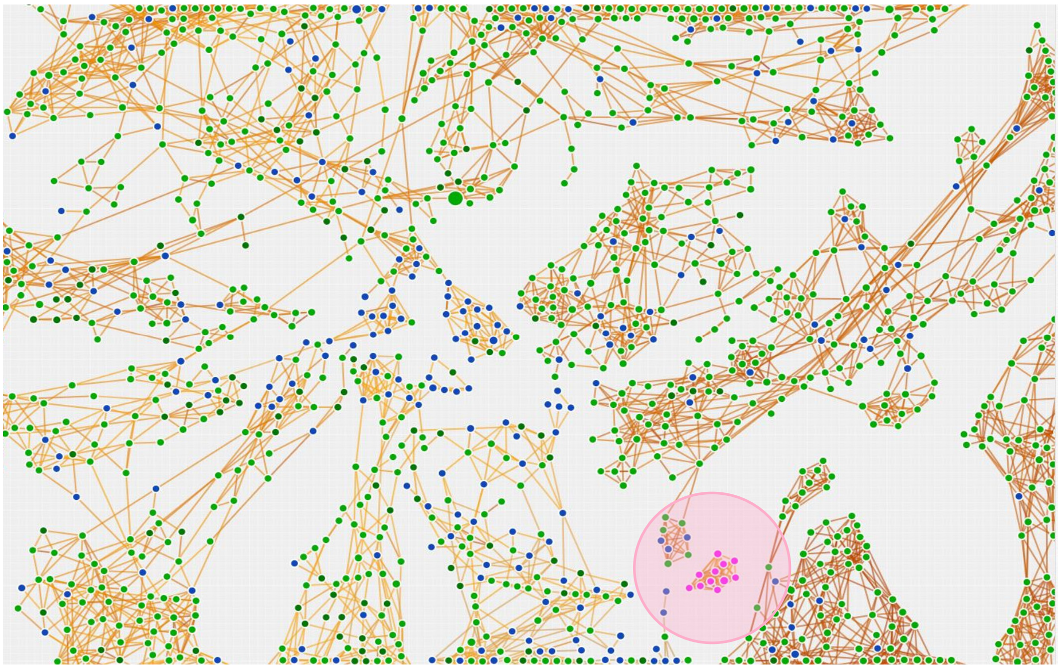

Figure S5. Molecular networking of the target food supplements highlighted the cyanotoxin cluster and its differentiation among the identified compounds.
